# Psychological induction of the child: Cognitive, emotional and behavioral diagnostic markers

**DOI:** 10.1192/j.eurpsy.2021.1897

**Published:** 2021-08-13

**Authors:** A. Kalashnikova

**Affiliations:** Laboratory Of Psychology, V. Serbsky National Medical Research Centre for Psychiatry and Narcology, Moscow, Russian Federation

**Keywords:** induced state, high-conflict divorces, children’s interests, forensic examination

## Abstract

**Introduction:**

One of the tasks of the forensic assessment of family disputes is to establish the fact that a child is set up by one parent against another.

**Objectives:**

Identification of diagnostic markers of psychological induced state in a child due to purposeful actions of a parent living together with him.

**Methods:**

A continuous one-step analysis of the results of forensic assessments on family disputes was carried out in respect of 48 girls and 67 boys aged 3 to 15 years (mean age 7.9 ± 4.5 years). The objective materials presented by the court were analyzed in comparison with the results of a structured interview. The statistical significance of any differences were evaluated using the non-parametric Mann-Whitney (U).

**Results:**

Persistent negative attitude to one of the parents was found in 14% of children. Markers of the induced state at the cognitive level were identified: negative semantic attitudes (U=477.1; p=0.014), distorted image of the rejected parent (U=509.5; p=0.023), transformation of memories (U=389.5; p=0.001). At the emotional level: persistent negative attitude to one of the parents when idealizing the second (U=371.1; p=0.001), emotional involvement of the child in the family conflict (U=556.6; p=0.048). At the behavioral level: declaring a stable set of stereotypical “adult” phrases (U=387.3; p=0.001), regressive behaviors and manifestations of stress in the presence of a rejected parent (U=601.5; p=0.04). Markers on all three levels must co-exist.

**Conclusions:**

There are diagnostic markers of the induced state in a child, which verify the forensic conclusion about the negative impact on his mental state of the parent-inducer.

**Disclosure:**

No significant relationships.

